# Sweet Taste Receptor Activation in the Gut Is of Limited Importance for Glucose-Stimulated GLP-1 and GIP Secretion

**DOI:** 10.3390/nu9040418

**Published:** 2017-04-22

**Authors:** Monika Y. Saltiel, Rune E. Kuhre, Charlotte B. Christiansen, Rasmus Eliasen, Kilian W. Conde-Frieboes, Mette M. Rosenkilde, Jens J. Holst

**Affiliations:** 1 NNF Center for Basic Metabolic Research and Department of Biomedical Sciences, Panum Institute, Faculty of Health and Medical Sciences, University of Copenhagen, Blegdamsvej, DK-2200, Copenhagen N, Denmark; monika.yosifova@sund.ku.dk (M.Y.S.); kuhre@sund.ku.dk (R.E.K.); cbchristiansen@sund.ku.dk (C.B.C.); rosenkilde@sund.ku.dk (M.M.R.); 2 Protein & Peptide Chemistry, Novo Nordisk A/S, Novo Nordisk Park, DK-2760 Måløv, Denmark; rael@Nanotech.dtu.dk (R.E.); kcf@novonordisk.com (K.W.C.-F.)

**Keywords:** sweet taste receptor, GLP-1, GIP, glucose, sucralose

## Abstract

Glucose stimulates the secretion of the incretin hormones: glucagon-like peptide-1 (GLP-1) and glucose-dependent insulinotropic peptide (GIP). It is debated whether the sweet taste receptor (STR) triggers this secretion. We investigated the role of STR activation for glucose-stimulated incretin secretion from an isolated perfused rat small intestine and whether selective STR activation by artificial sweeteners stimulates secretion. Intra-luminal administration of the STR agonists, acesulfame K (3.85% *w/v*), but not sucralose (1.25% *w/v*) and stevioside (2.5% *w/v*), stimulated GLP-1 secretion (acesulfame K: 31 ± 3 pmol/L vs. 21 ± 2 pmol/L, *p* < 0.05, *n* = 6). In contrast, intra-arterial administration of sucralose (10 mM) and stevioside (10 mM), but not acesulfame K, stimulated GLP-1 secretion (sucralose: 51 ± 6 pmol/L vs. 34 ± 4 pmol/L, *p* < 0.05; stevioside: 54 ± 6 pmol/L vs. 32 ± 2 pmol/L, *p* < 0.05, *n* = 6), while 0.1 mM and 1 mM sucralose did not affect the secretion. Luminal glucose (20% *w/v*) doubled GLP-1 and GIP secretion, but basolateral STR inhibition by gurmarin (2.5 µg/mL) or the inhibition of the transient receptor potential cation channel 5 (TRPM5) by triphenylphosphine oxide (TPPO) (100 µM) did not attenuate the responses. In conclusion, STR activation does not drive GIP/GLP-1 secretion itself, nor does it have a role for glucose-stimulated GLP-1 or GIP secretion.

## 1. Introduction

A high consumption of sugar may lead to a higher prevalence of unhealthy metabolic conditions, like diabetes type 2, obesity, and metabolic syndrome. Therefore, it is of great importance to understand how sugars are metabolized in the body and which feedback mechanisms regulate this process, and thus how it could be controlled or manipulated. As an alternative to sugars, artificial sweeteners have attracted considerable interest in the battle with obesity and diabetes, as they do not elevate blood glucose levels and are not a source of additional calories. Nevertheless, epidemiological studies show that their consumption may still be associated with weight gain and diabetes type 2 due to adaptive mechanisms [1,2], although randomized controlled trials, investigating the effects of sweeteners on body weight, show that they may reduce weight compared to groups consuming water [3,4].

In response to meal ingestion, several hormones that regulate blood sugar levels and appetite are secreted. The incretin peptide hormones, glucose-dependent insulinotropic hormone (GIP) and glucagon-like peptide 1 (GLP-1), secreted from the intestinal K- and L-cells, respectively, play an important role in this process, as they both stimulate insulin secretion, and GLP-1 suppresses appetite and slows gastric emptying [5]. 

Glucose is an efficacious stimulus for the secretion of both GLP-1 and GIP, stimulating secretory responses that are comparable to the response after the intake of an isocaloric mixed meal [6]. While it is well established that glucose stimulates the secretion of GIP and GLP-1, several different molecular sensors may be involved. A main driver for secretion appears to be glucose transportation through sodium-glucose dependent transporter 1 (SGLT1) [7,8,9,10,11,12], triggering hormone secretion by the depolarization of the K/L-cell plasma membrane and the opening of voltage sensitive calcium channels [7]. Electroneutral GLUT2-mediated uptake may potentiate glucose-stimulated GLP-1 secretion [7], potentially by the closure of the K_ATP_ channel upon intracellular glucose metabolism to ATP [7,13]. Another molecular sensor that has been suggested to be implicated in the secretory response is the sweet-taste receptor (STR). It is activated by glucose and other sweet-taste molecules, and is found not only in the taste buds, but also in the enteroendocrine K- and L-cells along the gastrointestinal tract, with the highest expression rates found in the proximal intestine [14,15,16,17].

The STR is a heterodimer, formed by the subunits T1R2 and T1R3, coupled to the G-proteins α-gustducin and/or transducin [18]. Around one fifth of the cells expressing α-gustducin co-express GLP-1 and GIP [16,19,20]. Depending on the ligand, receptor activation activates adenylate cyclase and the formation of cAMP (Gαs-coupling), or mobilizes calcium from intracellular stores (Gq-coupling) by the activation of phospholipase C (PLC) and inositol triphosphate (IP3) [21]. The increase in intracellular Ca2+ opens the nonselective transient receptor potential monovalent cation channel 5 (TRPM5), which plays a critical role in sweet, bitter, and umami taste signal transduction, leading to Na+ influx, depolarization of the cell, and eventual hormone or neurotransmitter secretion [22]. Natural sweeteners (sucrose, glucose, fructose) activate STR in the range of 100 mM, while less than 10 mM of the artificial sweeteners is sufficient for activation [18]. Studies on incretin secreting cell lines (derived from human and mouse gut carcinomas) have shown that STR activation by artificial sweeteners elicits GLP-1 secretion [16,17], and in humans, the inhibition of the gut sweet taste receptor by lactisole attenuated the glucose-stimulated GLP-1 secretion [19]. However, these findings remain inconclusive, as other studies found no effect of artificial sweeteners on GLP-1 secretion in humans [11,23,24,25,26,27] and rats (in vivo and using isolated perfused intestine preparations) [7,28]. The only studies in humans that found an increase in GLP-1 secretion after artificial sweetener consumption investigated combinations of artificial sweeteners in diet drinks or studied whether sweeteners potentiated glucose-stimulated GLP-1 secretion rather than driving secretion per se [29,30]. An important question, which none of the studies explored by means other than immunohistochemistry, is whether the sweet receptors are located apically or at the basolateral side of the L-cells, which might play a role in the lack of a response in vivo as artificial sweeteners, like sucralose, are incompletely absorbed.

The purpose of this study was to investigate the importance of sweet taste receptor activation for glucose-stimulated hormone secretion, with a primary focus on GLP-1 secretion and a secondary focus on GIP secretion. For this investigation, we used the isolated perfused rat small intestine, which is a physiological model, maintaining the enteroendocrine cells in their normal environment, polarized form with preserved vascular circulation and contact to normal neighbor cells, paracrine cells, and neurons, while the input and output from the organ was strictly controlled. Due to the preservation of natural polarity, this model also has the advantage that it can be used to investigate whether the stimulus has an effect on the apical or basolateral membrane of the cell.

## 2. Materials and Methods 

### 2.1. Perfusion of the Proximal Small Intestine 

Studies were performed with permission from the Danish Animal Experiments Inspectorate (2013-15-2934-00833) and the local ethics committee (EMED, P-15-408) in accordance with the guidelines of the Danish legislation governing animal experimentation (1987) and the National Institutes of Health. The experimental method and protocol have been described elsewhere in detail [7]. In brief, male Wistar rats (weight: mean + SEM = 283 ± 28.8 g) were obtained from Janvier labs (Le Genest-Saint-Isle, France), and were housed two per cage on a 12:12 h light/dark cycle, with ad libitum access to standard chow and water. On the experimental day, animals (non-fasted) were anesthetized with a subcutane Hypnorm/Midazolam injection and placed on a 37 °C heating plate. The abdominal cavity was opened; the entire large intestine and the distal half of the small intestine was carefully removed, leaving 44 ± 5.2 cm of the upper small intestine in situ (approximately half of the entire small intestine). A plastic tube was inserted into the proximal part of the lumen and the intestinal content was carefully removed by flushing with isotonic saline (room temperature). Next, the lumen was perfused with saline at a steady flow of 0.5 mL/min. A catheter was placed in the superior mesenteric artery and the intestine was vascularly perfused with gassed perfusion buffer (95% O_2_−5% CO_2_) warmed to 37 °C at a rate of 7.5 mL/min. Perfusion effluent was collected each minute from a catheter inserted in the vena portae and samples were instantly transferred to ice and stored at −20°C until analysis. The perfusion buffer consisted of a Krebs-Ringer bicarbonate buffer supplemented with 0.1% BSA (albumin fraction V; Merck, cat. no. 1.12018.0500, Ballerup, Denmark), 3.5 mmol/L glucose, 5% dextran T-70 (to balance oncotic pressure; Pharmacosmos, Holbaek, Denmark), 5 mmol/L pyruvate, 10 µmol/L 3-Isobutyl-1-methylxanthine (IBMX) (Sigma-Aldrich, cat. no. 5879), fumarate, glutamate, and 2 mL/L of Vamin (cat. no. 11338; Fresenius Kabi, Uppsala, Sweden). The pH was adjusted with hydrochloric acid to 7.4–7.5. After the successful perfusion of the proximal small intestine, rats were sacrificed by cardiac perforation and the preparation was left to stabilize hormone secretion for approximately 30 min before the samples were collected. 

### 2.2. Perfusion Protocol and Test Substances

Each experimental protocol started with ten minutes of baseline collection (pre-stimulatory period), followed by a stimulation for ten minutes, either luminally or vascularly. The intestine was stimulated luminally with 20% (*w/v*) glucose (1.1 mol/L) (Sigma-Aldrich, cat. no. G8270), 1.25% (*w/v*) sucralose (31.4 mmol/L) (Sigma-Aldrich, cat. no. 69293), 3.85% (*w/v*) acesulfame K (191.3 mmol/L) (Sigma-Aldrich, cat. no. 04054), or 2.5% (*w/v*) stevioside (31.1 mmol/L) (Sigma-Aldrich cat. no. CDS020802). The concentrations of the sweeteners employed correspond to 50 times the sweetness of 20% (*w/v*) glucose, given that sucralose, acesulfame K, and stevioside are 800, 260, and 400 times sweeter, respectively [31,32], and are all far beyond their respective EC50 values (0.3 mM for sucralose, 1 mM for acesulfame K, and 0.1 mM for stevioside [33]). Therefore, all of the solutions maximally activated the receptor. Luminal test stimulants were dissolved in isotonic saline without the addition of detergents and were applied at an initial rate of 2.5 mL/min for the first three minutes, followed by a rate of 0.5 mL/min during the rest of the stimulation. After the stimulation, isotonic saline was infused in the same pattern to replace test stimulant solution and reestablish baseline conditions. Vascular test stimulants (acefulfame K, sucralose, and stevioside) were dissolved in perfusion buffer to a final concentration of 10 mM (for sucralose also concentration 1 mM and 0.1 mM). To allow hormone secretion to return to the baseline after test stimulant application, stimulant applications were separated by 15 min. In separate experiments, the murine sweet taste receptor inhibitor gurmarin [34] (2.5 µg/mL) (provided by Rasmus Eliasen, synthetized at Novo Nordisk A/S as previously described [35]), was infused on the vascular side mixed with sucralose (10 mM) or simultaneously with glucose 20% (*w/v*) on the luminal side. Before the stimulation, the sweet taste receptor was primed with gurmarin for 15 min to ensure full inhibition at the time of test substance application. Using the same protocol, the TRPM5 channel was inhibited by the vascular administration (100 µM) of triphenylphosphine oxide (TPPO) (IC_50_ = 30 µM) (Sigma-Aldrich, cat. No T84603) [36]. To dissolve TPPO, it was mixed in 1% dimethyl sulfoxid solution and perfusion buffer. At the end of each experiment, the intestine was stimulated vascularly with bombesin (10 nM) (a potent GLP-1 secretagogue), to ensure that the preparation was viable until the end of the experiments. The perfusion pressure and effluent output were measured continuously, in order to evaluate the gut integrity and health during the experiment. Since all experiments were conducted with constant perfusion flow rates, all effluent hormone or metabolite concentrations correspond to the total secretion or absorption rates.

### 2.3. Hormone Secretion Analysis

GLP-1 concentrations in the venous effluents were analyzed using an in-house radioimmunoassay (RIA), employing a rabbit antiserum directed against the C-terminus of GLP-1 (code no. 89390), thus reacting with all amidated forms of GLP-1 (1-36NH2, 7-36NH2 and 9-36NH2).

GIP concentrations were quantified with an ELISA assay for the rat total GIP (Millipore, Merck, cat. no. EZRMGIP-55K), following the provided instructions.

### 2.4. Statistical Analysis

Changes in the hormone secretion were assessed by comparing the mean concentrations during the stimulation period with the baseline mean concentrations calculated as a mean of five consecutive one-minute observations before the stimulation and five one-minute observations before the start of the following stimulation. The stimulation period was defined as the start of the stimulant application until the end of the application. Statistical significance was assessed by one-way ANOVA, followed by Bonferroni post hoc analysis or a paired *t*-test, in the case that only two groups were compared. In the experiments where responses to stimuli with or without the presence of an inhibitor were compared, differences were assessed by a two-way paired *t*-test. Statistical analysis was performed using GraphPad Prism 7 (La Jolla, CA, USA). Data are expressed as averaged means ± SEM. *p* < 0.05 was considered significant.

## 3. Results

### 3.1. Glucose-Induced Incretin Secretion

#### 3.1.1. Glucose Stimulates GLP-1 and GIP Secretion from the Perfused Rat Small Intestine

**Figure 1:**
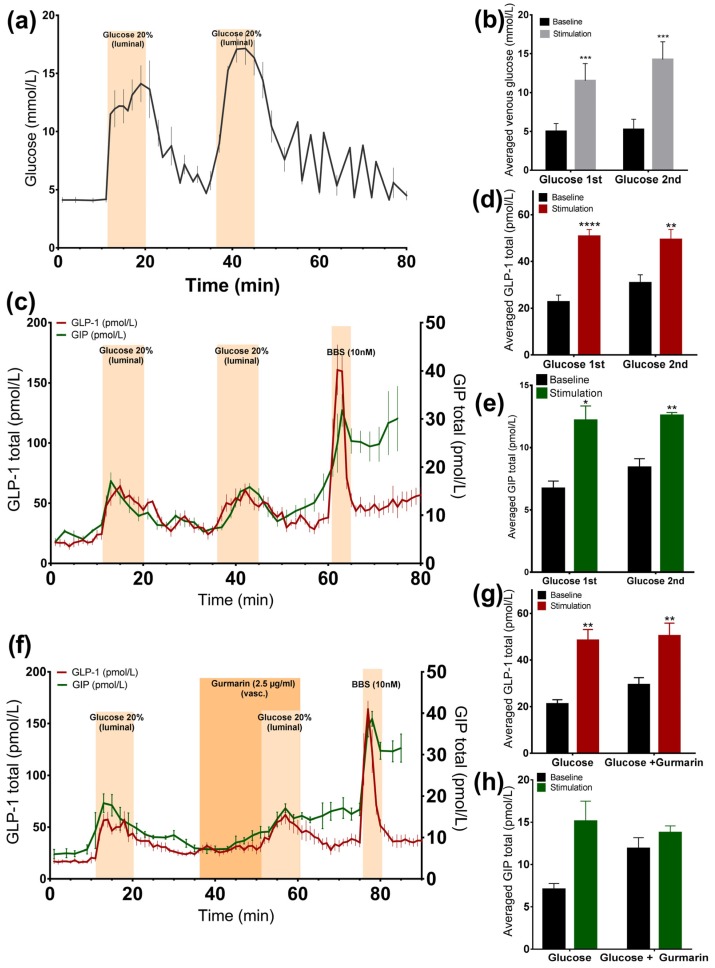
Glucose is a potent GLP-1 and GIP secretagogue and it is rapidly absorbed in the upper small intestine. Data is shown as averaged mean values ± SEM. (**a**) Glucose output in venous effluents in response to administration of luminal glucose. (**b**) Comparison between the venous glucose output upon glucose (stimulation) or saline (baseline). (**c**) GLP-1 (red line) and GIP (green line) secretion in response to luminal glucose. (**d**,**e**) Comparison between total GLP-1 or GIP output caused by glucose or saline. (**f**) GLP-1 (red line) and GIP (green line) secretion by luminal glucose administration and inhibition of the sweet taste receptor. (**g**,**h**) Comparison between the mean values of GLP-1 or GIP output upon glucose infusion ± gurmarin and saline. * *p* < 0.05, ** *p* < 0.01, *** *p* < 0.001. BBS bombesin; GLP-1 glucagon-like peptide 1; GIP glucose-dependent insulinotropic peptide.

Luminally administered glucose 20% (*w/v*) was rapidly absorbed, increasing venous effluent glucose concentrations by two to three times compared to the baseline (*glucose 1st*: 11.5 ± 0.9 mmol/L vs. *baseline 1st*: 5 ± 0.4 mmol/L, *p* < 0.001; *glucose 2nd*: 14.2 ± 0.9 mmol/L vs. *baseline 2nd*: 5.2 ± 0.5 mmol/L, *p* < 0.001, *n* = 6, Figure 1a,b). At the same time, we observed a doubling in the GLP-1 (*glucose 1st*: 50.7 ± 2.9 pmol/L vs. *baseline 1st*: 22.6 ± 3 pmol/L, *p* < 0.0001; *glucose 2nd*: 49.3 ± 4.4 pmol/L vs. *baseline 2nd*: 30.8 ± 3.5 pmol/L, *p* < 0.01, *n* = 6, Figure 1c,d) and GIP secretion rate (*glucose 1st*: 12.2 ± 1.2 pmol/L vs. *baseline 1st*: 6.7 ± 0.6 pmol/L, *p* < 0.05; *glucose 2nd*: 12.6 ± 0.2 pmol/L vs. *baseline 2nd*: 8.4 ± 0.7 pmol/L, *p* < 0.01, *n* = 6, Figure 1c,e), and in both cases, the second secretory response was not significantly different from the first (GLP-1 *p* = 0.55; GIP *p* = 0.73). GLP-1 and GIP showed a similar dynamic of secretion. In all experiments, a robust GLP-1 response to bombesin, administered at the end of the experiments (61–65 min), was observed, indicating that the experimental model was working until the end of the protocols (Figure 1c).

#### 3.1.2. Inhibition of the Murine Sweet Taste Receptor Does Not Change Glucose-Induced GLP-1 and GIP Secretion

In another series of experiments, the murine sweet taste receptor antagonist gurmarin was administered 15 min before and concomitantly with glucose (20% *w/v*). The inhibition of the sweet taste receptor did not attenuate glucose-induced GLP-1 secretion compared to glucose administration alone (Figure 1f), as the responses did not differ significantly (*p* = 0.73). Glucose increased GLP-1 secretion close by nearly two times, independently of whether it was added alone or concomitantly with gurmarin (*glucose*: 48.5 ± 4.6 pmol/L vs. *baseline 1*: 21.1 ± 1.9 pmol/L, *p* < 0.01; *glucose + gurmarin*: 50.3 ± 5.5 pmol/L vs. *baseline 2*: 29.4 ± 3 pmol/L, *p* < 0.01, *n* = 6, Figure 1f,g). The glucose-stimulated GIP response was also not significantly altered due to the presence of gurmarin (*p* = 0.58) (*glucose*: 15.1 ± 2.4 pmol/L vs. *baseline 1*: 7.1 ± 0.7 pmol/L, *p* = 0.13; *glucose + gurmarin*: 13.8 ± 0.8 pmol/L vs. *baseline 2*: 11.9 ± 1.3 pmol/L, *p* = 0.24, *n* = 4, Figure 1f,h). 

### 3.2. Arificial Sweeteners and Incretin Secretion

#### 3.2.1. Luminal Administration of Acesulfame K, but Not Sucralose or Stevioside, Stimulates GLP-1, but Not GIP Secretion

**Figure 2:**
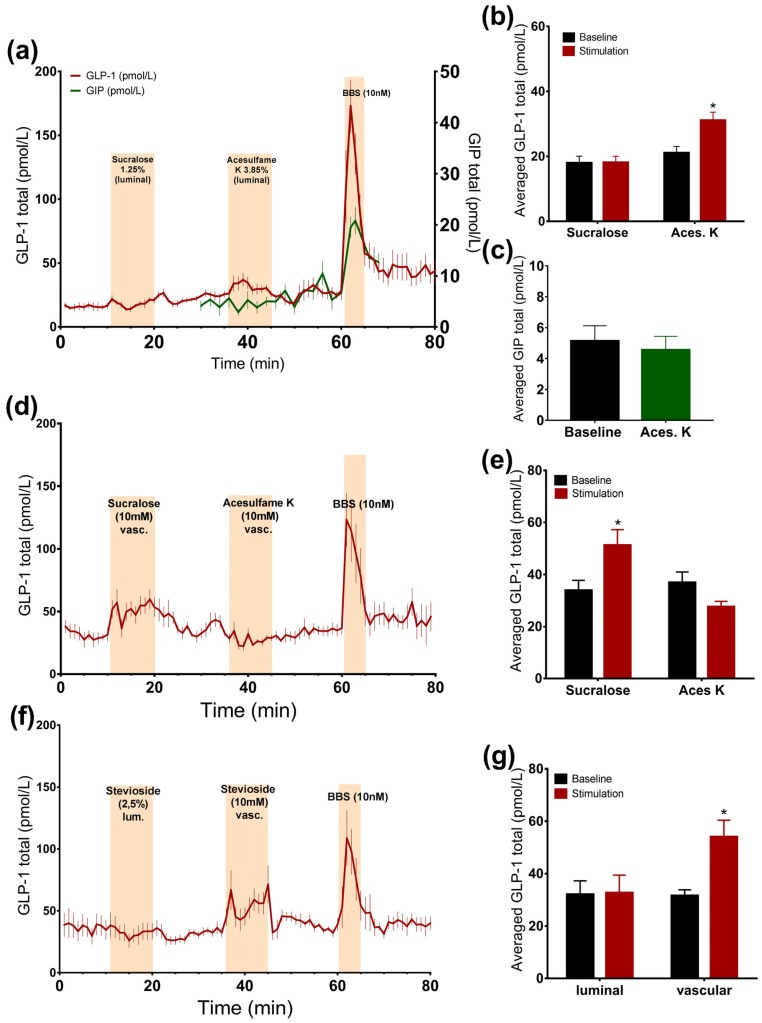
Effects of artificial sweeteners on incretin secretion. Data is shown as averaged mean values ± SEM (**a**) GLP-1 (red line) and GIP (green line) secretion in response to luminal sucralose and acesulfame K (**b**,**c**) Comparison between total GLP-1 or GIP output caused by luminal artificial sweeteners (stimulation) and saline (baseline) (**d**) GLP-1 secretion in response to vascular sucralose and acesulfame K (**e**) Comparison between the mean values of total GLP-1 secretion caused by vascular artificial sweeteners and saline (**f**) GLP-1 output in response to luminal and vascular stevioside administration (**g**) Comparison between the mean values of GLP-1 secretion upon liminal and vascular stevioside and saline * *p* < 0.05, ** *p* < 0.01, *** *p* < 0.001. BBS bombesin; GLP-1 glucagon-like peptide 1; GIP glucose-dependent insulinotropic peptide.

Intra-luminal administration of acesulfame K at a concentration that is thought to activate the sweet taste receptor resulted in a small, but significant, 1.5-fold increase in the GLP-1 concentrations (*acesulfame K*: 31.1 ± 2.5 pmol/L vs. *baseline*: 21.1 ± 2 pmol/L, *p* < 0.05, *n* = 6, Figure 2a,b), whereas it did not affect GIP secretion (*acesulfame K*: 4.6 ± 0.9 pmol/L vs. *baseline*: 5.1 ± 1 pmol/L, *p* = 0.53, *n* = 5, Figure 2a,c). Luminal infusion of sucralose and stevioside in matched concentrations with respect to sweetness, on the other hand, did not elevate the secretion of GLP-1 (Figure 2a,b,f,g).

#### 3.2.2. Vascular Administration of Sucralose and Stevioside, but Not Acesulafme K, Stimulates GLP-1 and GIP Secretion

**Figure 3:**
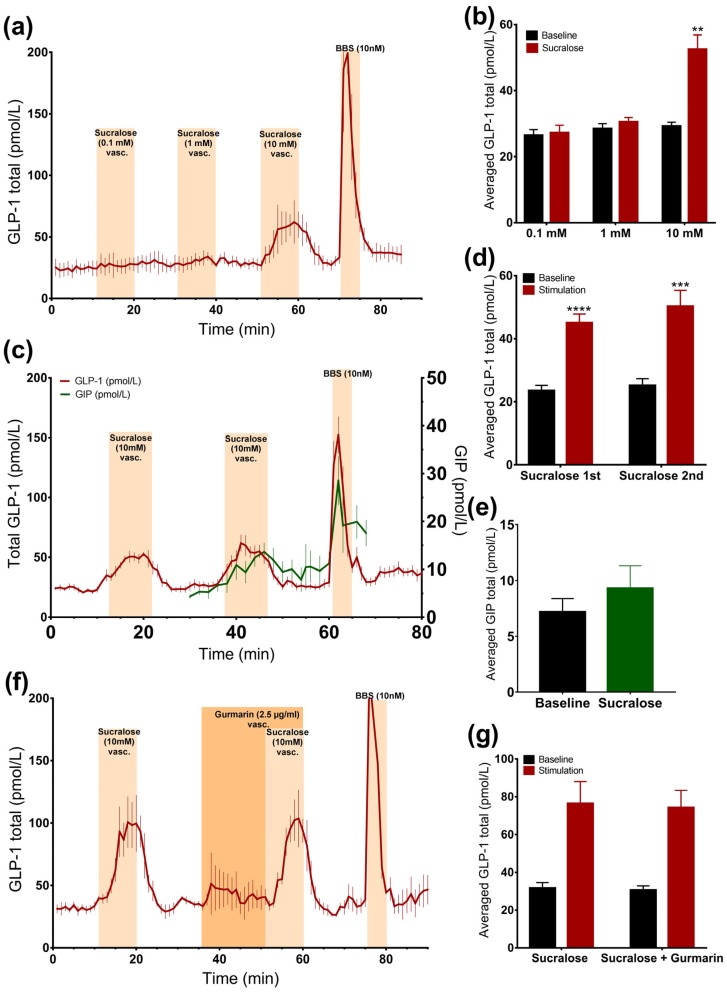
Effects of sucralose on the secretion of GLP-1 and GIP. Data is shown as averaged mean values ± SEM (**a**) GLP-1 secretion in response to vascular sucralose at 0.1 mM, 1 mM, and 10 mM concentrations (**b**) Comparison between the mean values of total GLP-1 output caused by vascular sucralose in increasing concentration (stimulation) and saline (baseline) (**c**) GLP-1 (red line) and GIP (green line) secretion in response to vascular administration of sucralose (**d**,**e**) Comparison between the mean values of total GLP-1 or GIP output caused by vascular sucralose and saline (**e**) GLP-1 secretion in response to vascular administration of sucralose ± gurmarin (**f,g**) Comparison between the mean values of GLP-1 output upon sucralose ± gurmarin * *p* < 0.05, ** *p* < 0.01, *** *p* < 0.001. BBS bombesin; GLP-1 glucagon-like peptide 1; GIP glucose-dependent insulinotropic peptide.

Intra-arterial administration of sucralose and stevioside significantly stimulated GLP-1 secretion (*sucralose*: 51.3 ± 5.9 pmol/L vs. *baseline*: 33.9 ± 3.9 pmol/L, *p* < 0.05, *n* = 6; *stevioside*: 54 ± 6.4 pmol/L vs. *baseline*: 31.5 ± 2.3 pmol/L, *p* < 0.05, *n* = 6, Figure 2d–g). However, vascular acesulfame K did not change the secretion rate (Figure 2d,e). The results were unchanged by the administration of acesulfame K before sucralose, suggesting that it is not the unavailability of the receptor that is responsible for the lack of the response to acesulfame K (data not shown). Sucralose slightly elevated GIP concentrations, although not significantly (*sucralose*: 9.3 ± 2 pmol/L vs. *baseline*: 7.2 ± 1.2 pmol/L, *p* = 0.09, *n* = 5, Figure 3c,e).

#### 3.2.3. Sucralose Only Stimulates GLP-1 Secretion at High Doses

In order to evaluate whether sucralose also stimulates GLP-1 secretion from the vascular side at doses lower than 10 mM, we stimulated the gut with 10 mM, 1 mM, or 0.1 mM sucralose in a separate line of experiments. Only 10 mM sucralose stimulated secretion (Figure 3a,b). 

Stimulating the intestine with 10 mM of sucralose resulted in two secretory responses that did not differ (*p* = 0.25) (*sucralose 1*: 45 ± 2.8 pmol/L vs. *baseline 1*: 23.5 ± 1.7 pmol/L, *p* < 0.0001; *sucralose 2*: 50.3 ± 5 pmol/L vs. *baseline 2*: 25.2 ± 2.2 pmol/L, *p* < 0.001, *n* = 6, Figure 3c,d), which allowed us to test whether the sucralose-induced GLP-1 secretion could be inhibited by gurmarin.

#### 3.2.4. Inhibition of the Murine Sweet Taste Receptor Does Not Eliminate Sucralose-Induced GLP-1 secretion

The inhibition of the sweet taste receptor before and together with sucralose administration (10 mM) did not affect GLP-1 secretion compared to sucralose alone, as the baseline-subtracted responses were not significantly different, and sucralose increased GLP-1 levels by around two times, independently of gurmarin administration (*sucralose*: 76.4 ± 11.7 pmol/L vs. *baseline 1*: 31.6 ± 3 pmol/L, *p* = 0.09; *sucralose + gurmarin*: 74.2 ± 9.2 pmol/L vs. *baseline 2*: 30.6 ± 2.3 pmol/L, *p* = 0.08, *n* = 3, Figure 3f,g).

### 3.3. Inhibition of TRPM5-Channel Does Not Attenuate Glucose or Sucralose-Stimulated GLP-1 Secretion

#### 3.3.1. Glucose-Induced GLP-1 Secretion Was not Reduced, but Increased, by Inhibition of TRPM5 Channels

**Figure 4:**
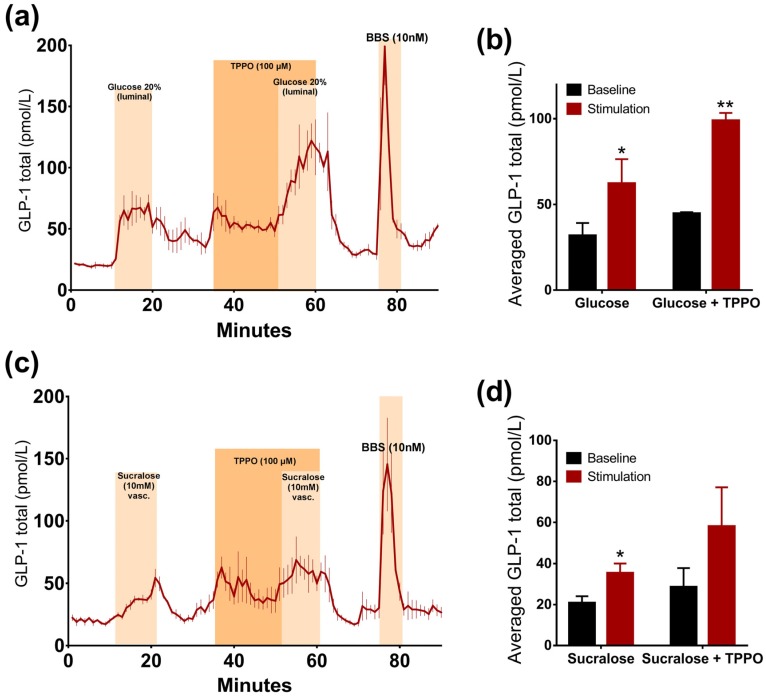
TRPM5 channel is not involved in glucose- and sucralose-stimulated GLP-1 secretion. Data is shown as averaged mean values values ± SEM (**a**) GLP-1 secretion in response to luminal glucose alone or in the presence of TPPO (**b**) Comparison between the mean values of total GLP-1 output caused by luminal glucose ± TPPO (stimulation) and saline (baseline) (**c**) GLP-1 secretion in response to vascular administration of sucralose alone or in presence of TPPO (**d**) Comparison between the mean values of total GLP-1 output caused by vascular sucralose ± TPPO and saline * *p* < 0.05, ** *p* < 0.01, *** *p* < 0.001. BBS bombesin; GLP-1 glucagon-like peptide 1; GIP glucose-dependent insulinotropic peptide.

The inhibition of the TRPM5 ion channel by TPPO did not lead to a sweet taste receptor mediated reduction in GLP-1 secretion, but on the contrary, led to a significant increase in glucose-stimulated GLP-1 secretion (*p* = 0.04). Due to an increase in basal secretion after the administration of TPPO, differences were assessed by a comparison between the baseline-subtracted responses. Glucose alone doubled GLP-1 secretion, whereas the addition of TPPO increased it even more (*glucose*: 62.3 ± 8.2 pmol/L vs. *baseline 1*: 31.8 ± 4.3 pmol/L, *p* < 0.05; *glucose + TPPO*: 99 ± 2.5 pmol/L vs. *baseline 2*: 44.7 ± 0.4 pmol/L, *p* < 0.01, *n* = 3, Figure 4a,b).

#### 3.3.2. Sucralose-Induced GLP-1 Secretion Is not Changed by Inhibition of TRPM5 Channels

Sucralose-induced GLP-1 secretion was not reduced in the presence of the TRPM5 inhibitor TPPO. There was no significant difference between the responses to the administration of sucralose alone or sucralose concomitantly with TPPO, when comparing the baseline-subtracted responses (*p* = 0.11). Sucralose increased GLP-1 levels by around two times when infused alone or together with TPPO (*sucralose*: 35.4 ± 2.7 pmol/L vs. *baseline 1*: 20.7 ± 1.9 pmol/L, *p* < 0.05; *sucralose + TPPO*: 58 ± 11 pmol/L vs. *baseline 2*: 28.6 ± 2.3 pmol/L, *p* = 0.07, *n* = 3, Figure 4c,d). Interestingly, TPPO increased GLP-1 secretion when given alone (Figure 4).

## 4. Discussion

The involvement of the STR in glucose-stimulated GLP-1 and GIP secretion has been a matter of controversy during the last decade, with different studies suggesting that it may or may not be acutely involved in hormone secretion, glucose homeostasis, and appetite regulation [7,16,17,19,23,24,25,26,27,28,37]. This study shows that the ability of artificial sweeteners to stimulate GLP-1 secretion vascularly or luminally is sweetener-specific. Sucralose affected GLP-1 secretion when applied from the vascular side, whereas acesulfame K exclusively stimulated secretion when administrated in the intestinal lumen and only in pharmacological doses. This may explain why orally ingested artificial sweeteners do not trigger incretin hormones secretion in vivo [23,24,25,26,27,28], even though cell studies show the opposite [16,17].Vascular stimulation with sucralose in our perfusion model stimulated a secretory GLP-1 and GIP response, in agreement with the results from cell studies on GLUTag cells and NCI-H716 cells [16,17], but only at a concentration that must be considered to be pharmacological. However, as opposed to those studies, the secretory response in our model was not attenuated by STR inhibition with gurmarin. The fact that sucralose and stevioside triggered GLP-1 secretion when applied on the vascular side, but not on the luminal, suggests that the sweet taste receptor is either located on the basolateral membrane of the L-cells or that the response is triggered by other mechanisms. Nevertheless, we were not able to inhibit the sucralose-stimulated secretion by means of gurmarin or by inhibiting the TRPM5 channel, which is involved in the Gq-coupled taste sense and hormonal secretion [22]. Therefore, it seems that sucralose triggered hormone secretion by an STR-independent mechanism, which is consistent with our finding that sucralose at 0.1 and 1 mM did not affect secretion, albeit the sweetness of 1 mM is above the EC_50_-value of sucralose (0.3 mM) [33]. It may be argued that sucralose could stimulate GLP-1 and GIP secretion by acting on the bitter receptors, but as TRPM5 is also involved in other taste signal pathways, like umami and bitter [22], it is unlikely that sucralose acts on the L-cells by activating one of these receptors. Nevertheless, TRPM5 expression on the L-cells is low [38] and may not be as important for taste receptor signaling in the small intestine as it is for the taste cells on the tongue. Sucralose in concentrations of 10 mM and 100 mM, seem to exert toxic effects on IP3 production in GLUTag cells, which express the STR subunits [39] (data not shown), suggesting that it stimulates the secretion by another signal pathway. One could argue that the response seen following the vascular administration of sucralose and stevioside is triggered by high osmolarity. However, this is unlikely as intra-arterial acesulfame K at 10 mM in this study, and intra-arterial glucose at concentrations of 5 to 25 mM in one of our previous studies [7], did not affect GLP-1 secretion. 

Also, glucose-stimulated GLP-1 and GIP secretion was unaffected by gurmarin, standing in contrast to the finding from two human studies, where STR inhibtion (with lactisole—an inhibitor of the human STR) [19,37] attenuated glucose-stimulated GLP-1 secretion by about 50%. Surprisingly, the inhibition of the TRPM5 channel did not attenuate glucose-stimulated GLP-1 secretion, but even slightly increased it. This once again shows that it is probably not the STR-activated signal pathway that regulates secretion. As the IC_50_ of gurmarin on the STR was reported to be 0.62 µg/mL [40], and 0.5 µM gurmarin decreases the magnitude of sweet perception to sucralose by 70% on the taste buds in rats [41]. We anticipate that the lack of the effect of gurmarin in our experimental model is unlikely to be dose-related, as we applied the IC_50_ dose (2.5 µg/mL) four times, and this dose is comparable to the dose used by Margolskee and colleagues (3 µg/mL). Experiments with GLUTag cells showed that glucose increases intracellular calcium at a concentration of 100 mM, whereas gurmarin (5 µg/mL) attenuated glucose-stimulated intracellular calcium by around 10% (data not shown). This proves that gurmarin was bioactive. Nevertheless, we cannot exclude that the lack of an effect in our model is due to an unfavorable ratio between the inhibitor and the sweet-tasting agonists, as they were applied in high concentrations. 

Our finding that sucralose and stevioside did not stimulate GLP-1 secretion when applied into the lumen, even in a very high concentration, is in agreement with the results from human studies showing no effect of artificial sweeteners on GLP-1 secretion [23,24,25,27] or in vivo studies in rats [28]. Interestingly, intra-luminal acesulfame K increased GLP-1 secretion by 1.5-fold, which is in contrast to the results of a previous study by our group [7], but may be explained by the fact that in this study a five-times higher concentration was applied. Along this notation, the reason why luminal acesulfame K, but not sucralose and stevioside, stimulated GLP-1 secretion may be explained by the fact that a given dose of acesulfame K is completely recovered in the urine [42]. Therefore, it may be absorbed by the enterocytes in non-metabolized form and acts on the basolateral side of the L-cells in contrast to sucralose and stevioside, which are either incompletely absorbed or are metabolized by the bacteria in the colon and then absorbed [43,44]. In this way, acesulfame K may stimulate GLP-1 secretion by mechanisms similar to vascularly applied sucralose and stevioside, as the dose applied in the lumen corresponds to 191 mM. On the other hand, the lack of a response to acesulfame K when applied vascularly may be explained by the fact that it is less potent in activating the sweet taste receptor compared to sucralose and stevioside, and the applied dose was, therefore, insufficient.

Our study further shows that GLP-1 and GIP have similar dynamics of secretion and are therefore probably triggered by similar mechanisms, presumably involving the SGLT1 transporter as indicated by a number of studies from different groups using different experimental models [7,8,12]. 

The proximal part of the small intestine was chosen as a model in the current study, as glucose is almost entirely absorbed in the duodenum and jejunum [45]. Therefore, studying the effects of glucose on hormone secretion in the proximal small intestine gives a more physiologically relevant picture. In particular, in relation to this study, the relevant anatomical area of the intestine was used, as the sweet taste receptor is predominantly expressed in the duodenum and jejunum [14,15,16,17]. However, it may be a limitation that the very proximal part of the small intestine (the duodenum and the most proximal segment of the jejunum) was not included for technical reasons.

The advantage of the isolated perfused organ model, over in vivo studies, is that the confounding effects of several whole body parameters can be excluded. These include the effect of gastric emptying, the breakdown of various substances by whole body metabolism, and the impact of other regulatory hormones, while the physiological impact of specific compounds can be studied locally in the preserved organ. This gives the opportunity to establish a cause and effect relationship between the studied agent and the outcome. The risk of having the results influenced by indirect factors is minimal, since all compounds entering the system are strictly controlled. Therefore, in the present study, it could be established that sucralose and stevioside stimulate GLP-1 secretion when infused vascularly, but not when infused luminally. Compared to isolated cell studies, an alternative controlled system for the study of hormonal secretion, the perfused model also has the benefit that the natural polarity, inter-cell connection, and neural communication are preserved [46]. 

Limitations of the study include the limited translatability of the findings to humans in respect to artificial sweeteners, as they were applied in doses in the toxic range for humans. However, this was justified by the aim of the study to use them as a tool to explore sweet taste receptor functions, rather than to explore their physiological relevance. 

## 5. Conclusions

This study does not support the view that the activation of the gut sweet taste receptor plays a crucial role in the glucose-stimulated incretin secretion, as the inhibition of the receptor did not significantly reduce this secretion, suggesting that other mechanisms are fundamental for the GLP-1 secretion upon glucose administration. Our work suggests that the presence of pharmacological concentrations of sucralose and stevioside on the basolateral membrane may increase GLP-1 secretion. However, given the high concentrations needed to stimulate secretion, this finding is probably of limited clinical relevance. Future research should concentrate on compounds that target SGLT1- and GLUT2-mediated GLP-1 and GIP secretion, instead of compounds targeting the sweet taste receptor.
